# Supplementary figures and images for: Targeting IRE1α improves insulin sensitivity and thermogenesis and suppresses metabolically active adipose tissue macrophages in male obese mice
Source: eLife. 2025 Apr 17;13:RP100581. doi: 10.7554/eLife.100581 (PMC12005715; doi:10.7554/eLife.100581)

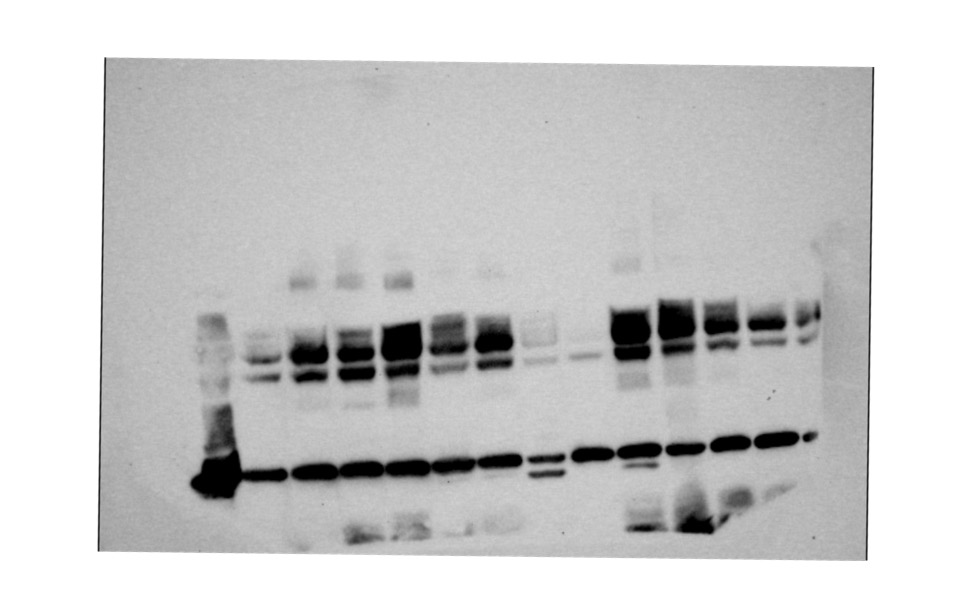

Supplement: Figure 1—source data 1. [file elife-100581-fig1-data1.zip › Figure 1-source data 1/GAPDH western blot.jpg]

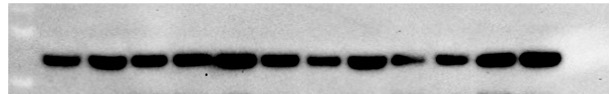

Supplement: Figure 1—source data 1. [file elife-100581-fig1-data1.zip › Figure 1-source data 1/tAKT for western blot.jpg]

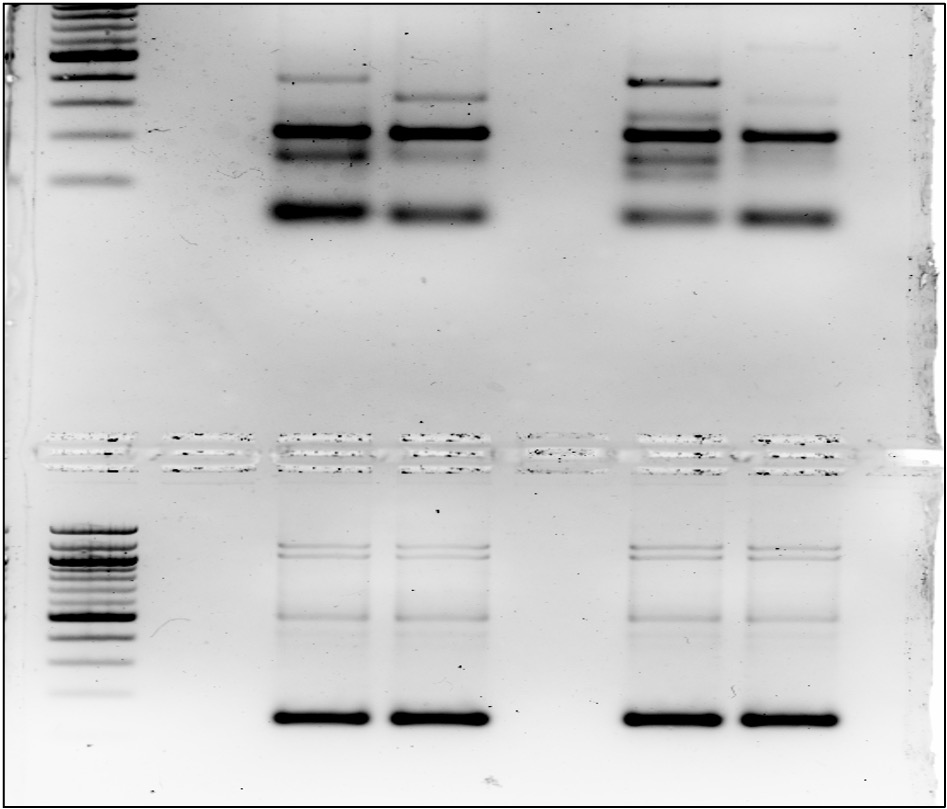

Supplement: Figure 1—source data 1. [file elife-100581-fig1-data1.zip › Figure 1-source data 1/Xbp1 splicing gel.jpg]

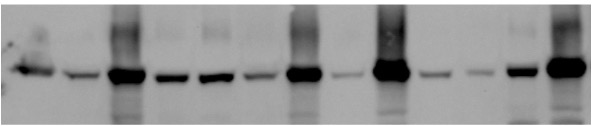

Supplement: Figure 1—source data 1. [file elife-100581-fig1-data1.zip › Figure 1-source data 1/pAKT for wester blot.jpg]

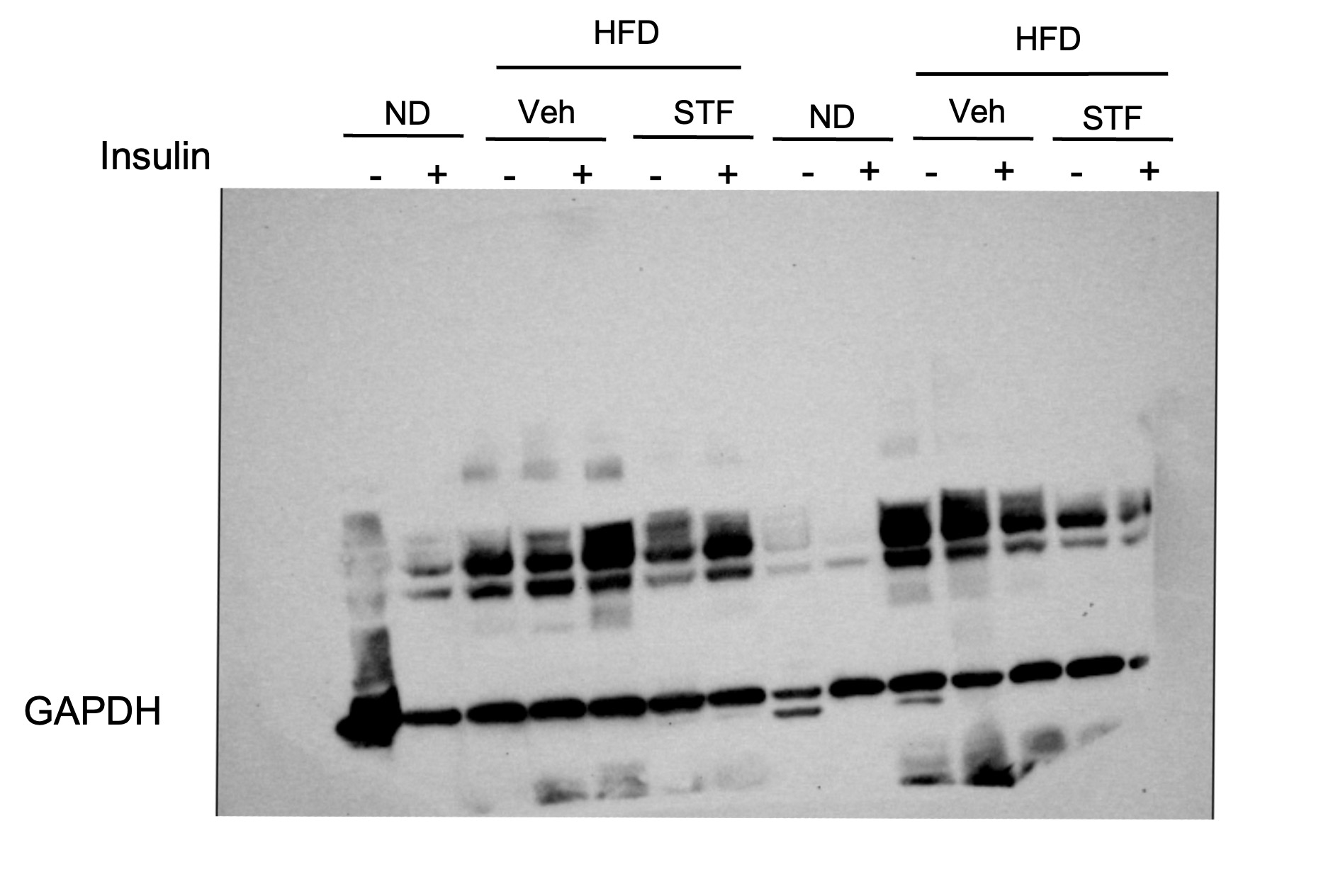

Supplement: Figure 1—source data 2. [file elife-100581-fig1-data2.zip › Figure 1-source data 2/GAPDH of western blot.jpg]

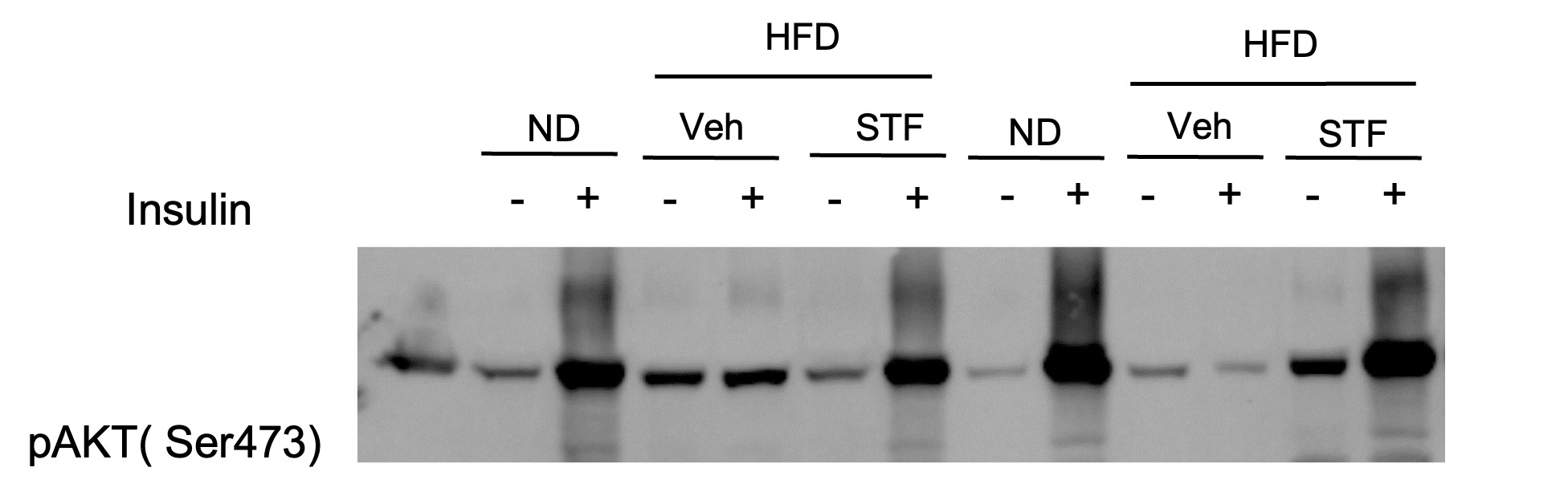

Supplement: Figure 1—source data 2. [file elife-100581-fig1-data2.zip › Figure 1-source data 2/pAKT expression of wester blot.jpg]

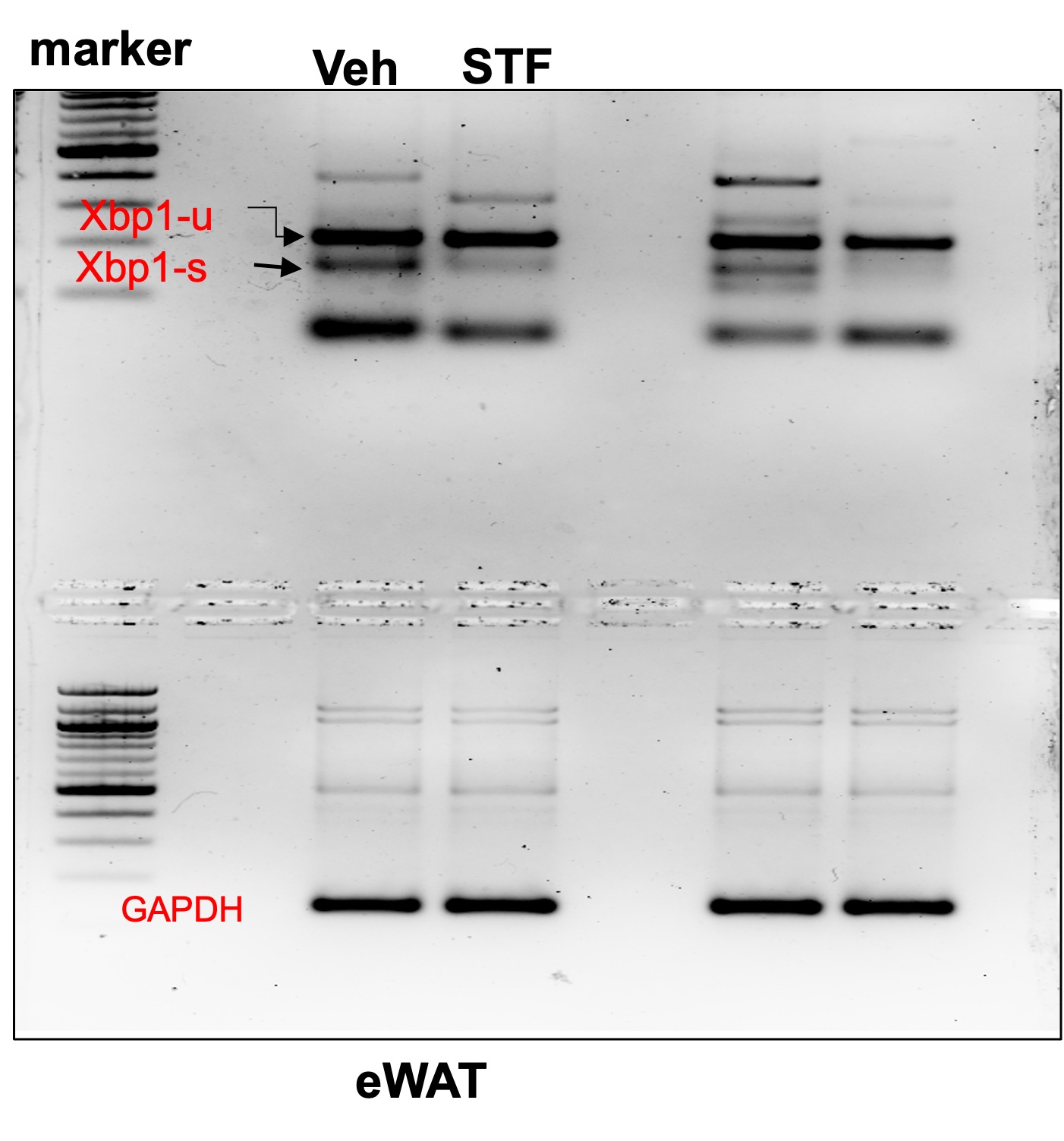

Supplement: Figure 1—source data 2. [file elife-100581-fig1-data2.zip › Figure 1-source data 2/xbp1 splicing in eWAT tissue.jpg]

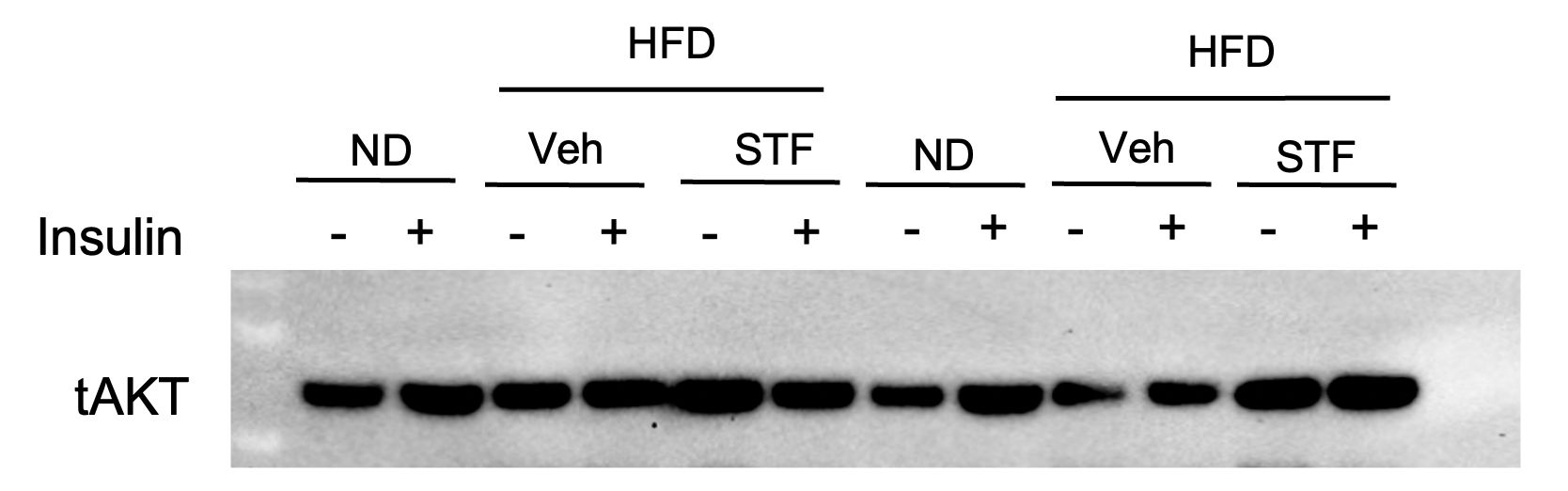

Supplement: Figure 1—source data 2. [file elife-100581-fig1-data2.zip › Figure 1-source data 2/tAKT expression of western blot.png]

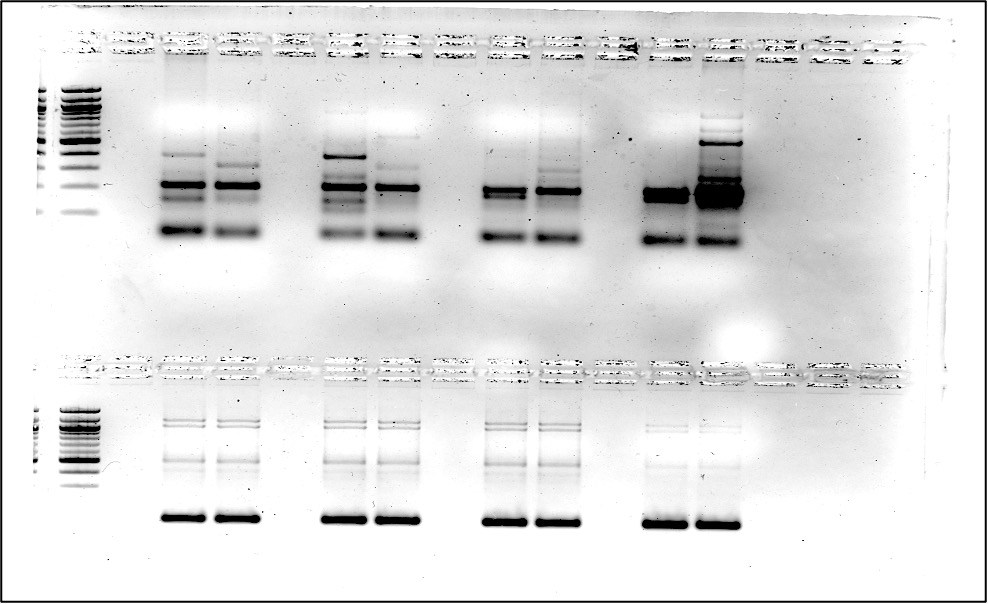

Supplement: Figure 1—figure supplement 1—source data 1. [file elife-100581-fig1-figsupp1-data1.zip › Figure 1-figure supplement 1-source data 1/Xbp1 splicing in iWAT.jpg]

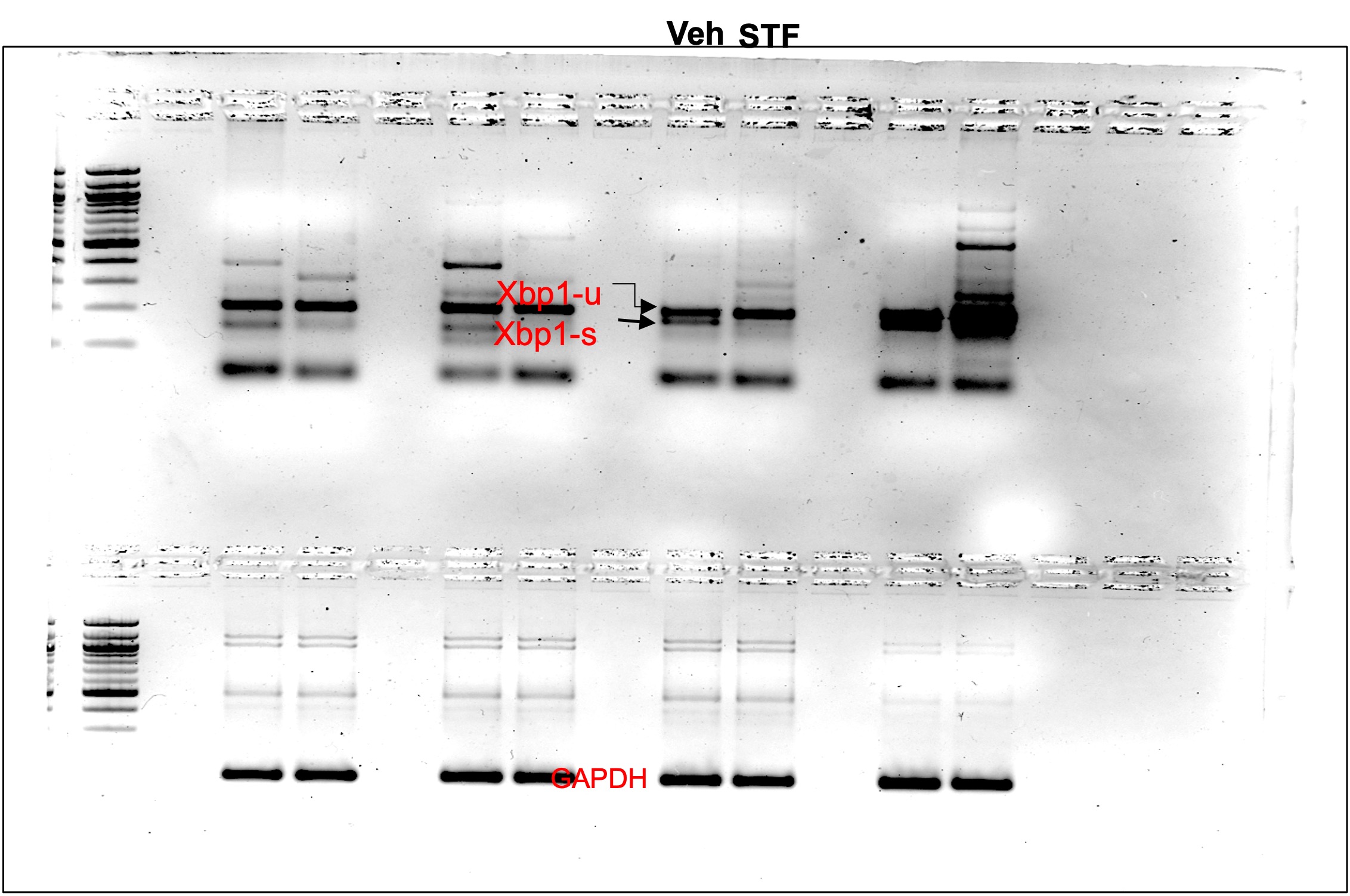

Supplement: Figure 1—figure supplement 1—source data 2. [file elife-100581-fig1-figsupp1-data2.zip › Figure 1-figure supplement 1-source data 2/Xbp1 splicing in iWAT.jpg]

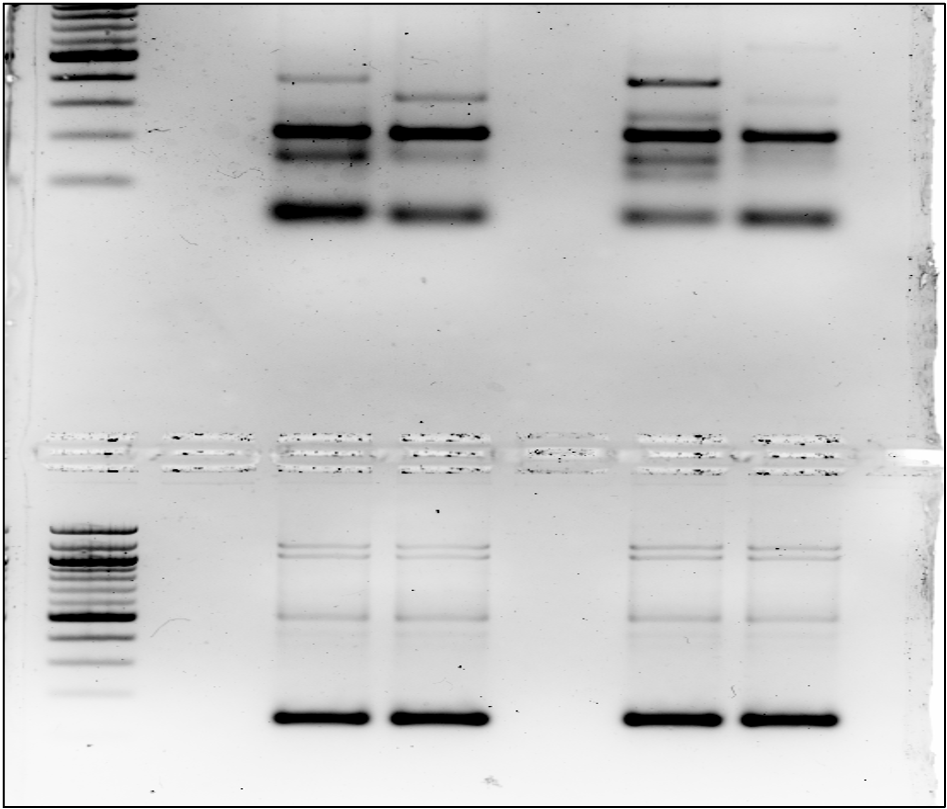

Supplement: Figure 1—figure supplement 1—source data 2. [file elife-100581-fig1-figsupp1-data2.zip › Figure 1-figure supplement 1-source data 2/Xbp1 splicing gel .png]

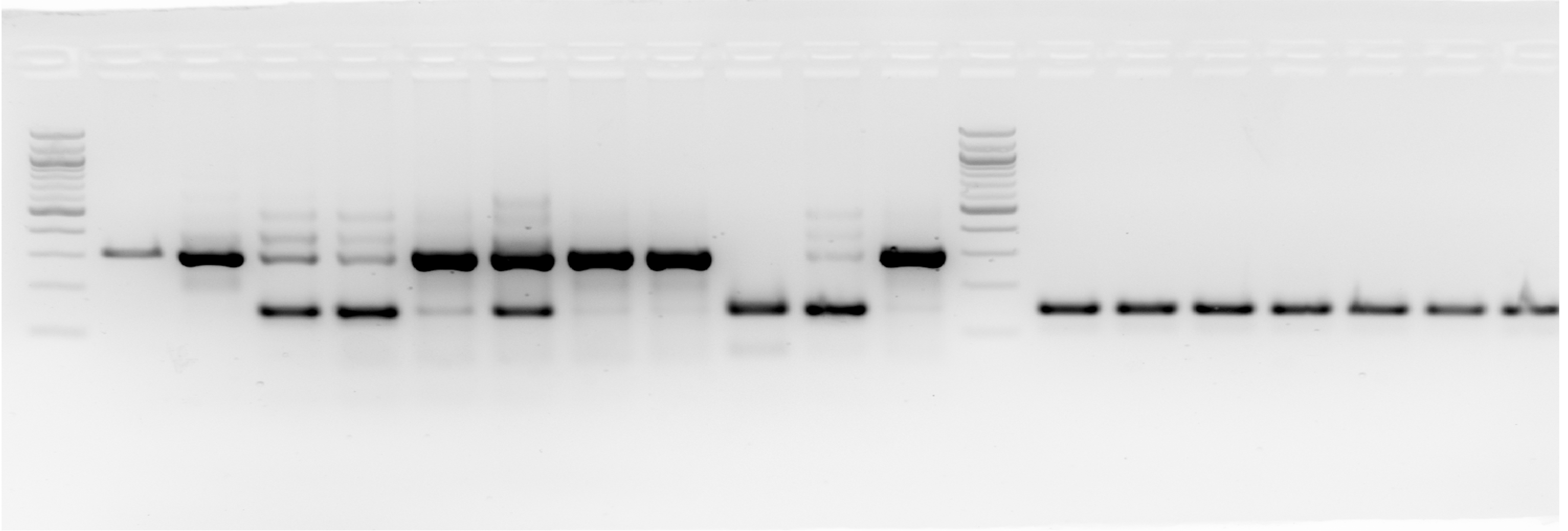

Supplement: Figure 5—figure supplement 1—source data 1. [file elife-100581-fig5-figsupp1-data1.zip › Figure 4-figure supplement 4-source data 1/GAPDH gels.png]

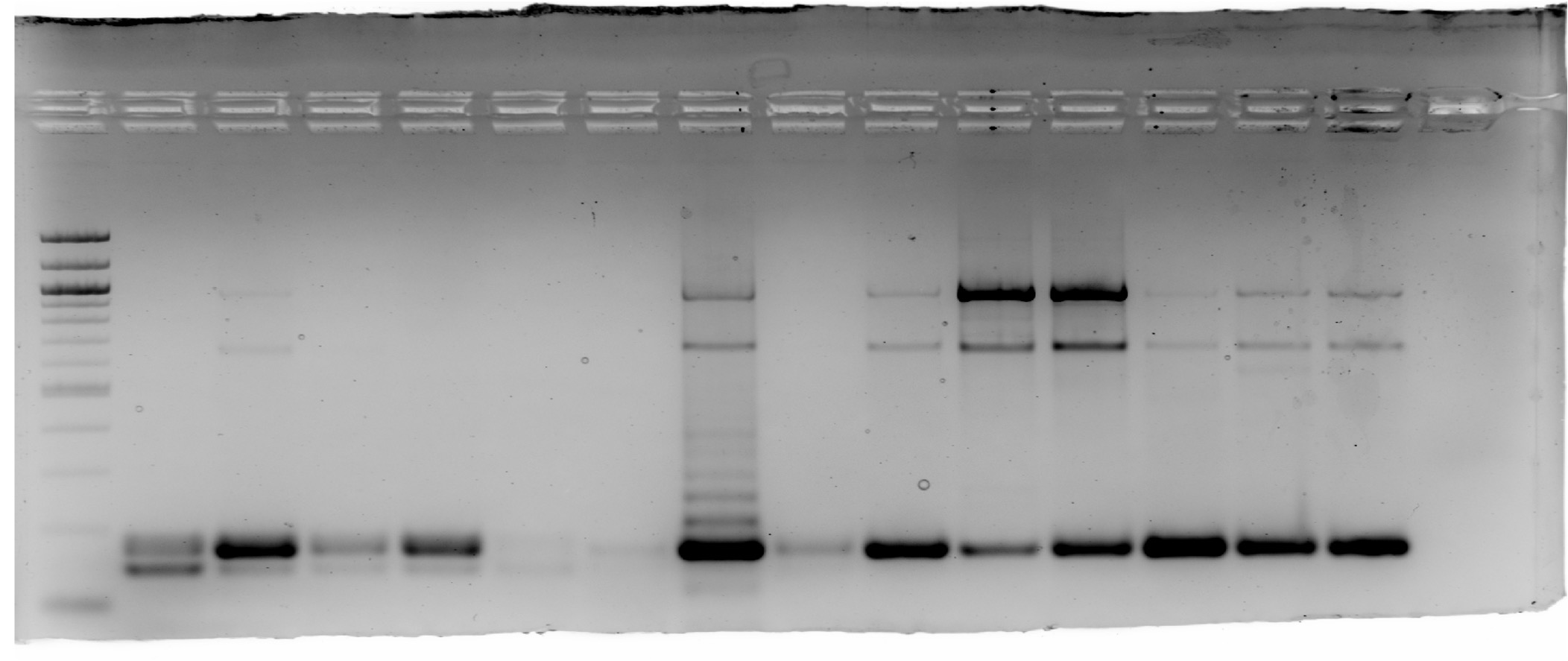

Supplement: Figure 5—figure supplement 1—source data 1. [file elife-100581-fig5-figsupp1-data1.zip › Figure 4-figure supplement 4-source data 1/Picture1.jpg]

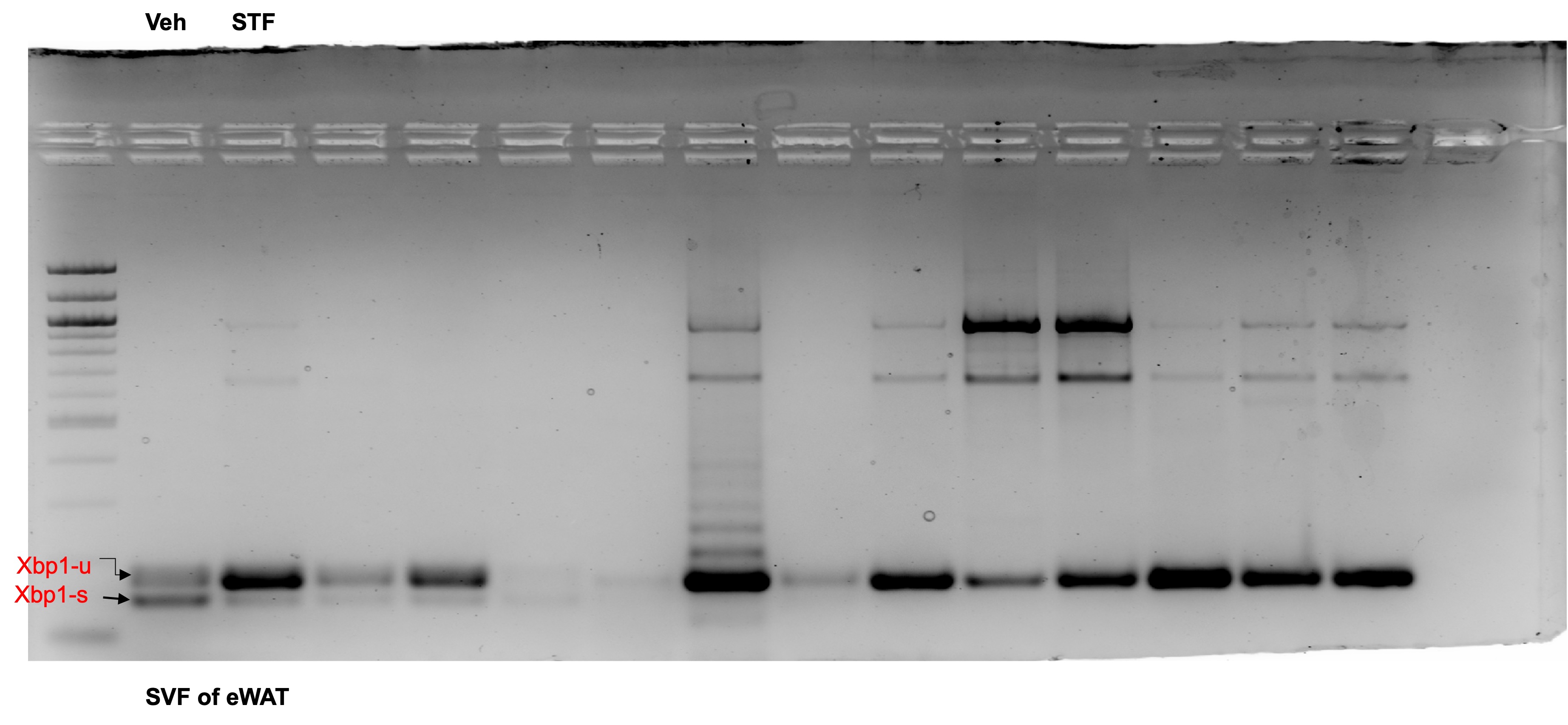

Supplement: Figure 5—figure supplement 1—source data 2. [file elife-100581-fig5-figsupp1-data2.zip › Figure 4-figure supplement 4-source data 2/Xbp1 splicing in eWAT SVF.jpg]

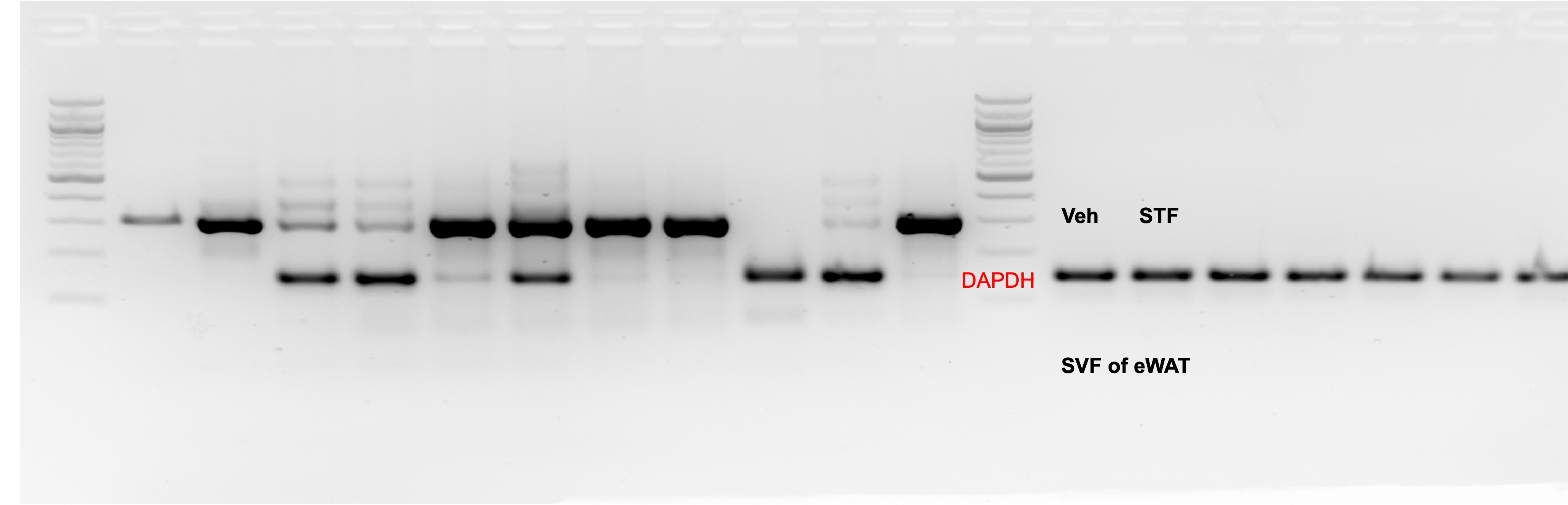

Supplement: Figure 5—figure supplement 1—source data 2. [file elife-100581-fig5-figsupp1-data2.zip › Figure 4-figure supplement 4-source data 2/GAPDH in eWAT SVF.jpg]
