# Supplementary material for: Targeting IRE1α improves insulin sensitivity and thermogenesis and suppresses metabolically active adipose tissue macrophages in male obese mice
Source: eLife. 2025 Apr 17;13:RP100581. doi: 10.7554/eLife.100581 (PMC12005715; doi:10.7554/eLife.100581)
Supplement: Supplementary file 1. — The total cell number and density of populations from CD11C+CD206- ATMs in the eWATs of mice with ND, HFD-Veh, or HFD-STF (a), CD11C-CD206- ATMs in the eWATs of mice with ND, HFD-Veh, or HFD-STF (b), and F4/80-CD11B- ATMs in the eWATs of DIO mice treated with Veh or STF (c). Data in a-c were obtained from 2 batches of 4 mice each and are the mean ± SEM. ∗P<0.05, ∗∗P<0.01, and ∗∗∗P<0.001. [file elife-100581-supp1.docx]

Supplementary File 1a

| **F4/80^+^CD11B^+^CD11C^+^CD206^-^** | | |
| --- | --- | --- |
|  | Total cell#/eWAT/mouse | Cell#/g of eWAT/mouse |
| ND | 0.12 ± 0.11 x10^4^ | 0.25 ± 0.23 x10^4^ |
| HFD-Veh | 1.43 ± 0.79 x10^5^ | 0.74 ± 0.37 x10^5^ |
| HFD-STF | 0.65 ± 0.43 x10^5^ | 0.28 ± 0.17 x10^5^ |
| HFD-Veh/ND: Fold (P value) | 118.61 (0.036) | 30.27 (0.027) |
| HFD-STF/HFD-Veh: Fold (P value) | 0.45 (0.20) | 0.38 (0.12) |

Supplementary File 1b

| **F4/80^+^CD11B^+^CD206^-^CD11C^-^** | | |
| --- | --- | --- |
|  | Total cell#/eWAT/mouse | Cell#/g of eWAT/mouse |
| ND | 0.42 ± 0.42 x10^4^ | 0.84 ± 0.83 x10^4^ |
| HFD-Veh | 0.71 ± 0.13 x10^5^ | 0.38 ± 0.038 x10^5^ |
| HFD-STF | 1.30 ± 1.03 x10^5^ | 0.56 ± 0.41 x10^5^ |
| HFD-Veh/ND: Fold (P value) | 16.97 (0.038) | 4.54 (0.084) |
| HFD-STF/HFD-Veh: Fold (P value) | 1.83 (0.63) | 1.46 (0.71) |

Supplementary File 1c

| **F4-80^-^CD11B^-^** | | |
| --- | --- | --- |
|  | Total cell#/eWAT/mouse | Cell#/g of eWAT/mouse |
| HFD-Veh | 7.04 ± 1.17 x10^5^ | 3.78 ± 0.33 x10^5^ |
| HFD-STF | 9.28 ± 1.57 x10^5^ | 4.32 ± 0.20 x10^5^ |
| HFD-STF/HFD-Veh: Fold (P value) | 1.32 (0.37) | 1.14 (0.29) |
