# Supplementary material for: Targeting IRE1α improves insulin sensitivity and thermogenesis and suppresses metabolically active adipose tissue macrophages in male obese mice
Source: eLife. 2025 Apr 17;13:RP100581. doi: 10.7554/eLife.100581 (PMC12005715; doi:10.7554/eLife.100581)
Supplement: Supplementary file 2. — The percentage, total cell number, and density of populations from CD9+CD63+ ATMs in the eWATs of mice with ND, HFD-Veh, or HFD-STF. Data were obtained from 2 batches of 4 mice each and are the mean ± SEM. ∗P<0.05, ∗∗P<0.01, and ∗∗∗P<0.001. [file elife-100581-supp2.docx]

Supplementary File 2

| **F4-80^+^CD11B^+^CD9^+^CD63^+^** | | | |
| --- | --- | --- | --- |
|  | Percentage | Total cell#/eWAT/mouse | Cell#/g of eWAT/mouse |
| ND | 18.00 ± 5.40 | 0.38 ± 0.29 x10^4^ | 0.87 ± 0.48 x10^4^ |
| HFD-Veh | 65.90 ± 1.50 | 9.42 ± 0.78 x10^5^ | 5.09 ± 0.011 x10^5^ |
| HFD-STF | 49.35 ± 9.65 | 3.65 ± 0.08 x10^5^ | 1.74 ± 0.25 x10^5^ |
| HFD-Veh/ND: Fold (P value) | 3.66 (0.013) | 245.23 (0.0069) | 58.86 (9.59E-05) |
| HFD-STF/HFD-Veh: Fold (P value) | 0.75 (0.23) | 0.39 (0.018) | 0.34 (0.0057) |
