# Supplementary material for: Targeting IRE1α improves insulin sensitivity and thermogenesis and suppresses metabolically active adipose tissue macrophages in male obese mice
Source: eLife. 2025 Apr 17;13:RP100581. doi: 10.7554/eLife.100581 (PMC12005715; doi:10.7554/eLife.100581)
Supplement: Supplementary file 3. [file elife-100581-supp3.docx]

|  |  |  |  |  |
| --- | --- | --- | --- | --- |
| **Gene** | **Accession Number** | **Forward Primer** | **Reverse Primer** |  |
| *Gapdh* | NM_008084 | AAAGACTGGAGCCCCACACTCTAC | ATCCCGTATTTCACCTCTGCTTC |  |
| *Xbp1s* | NM_013842 | GAG TCC GCA GCA GGT | GTG TCA GAG TCC ATG |  |
| *Xbp1u* | NM_013842 | CGCAGACTGCTCGAGATAGA | CCACAAGGCCGTGAGTTTTC |  |
| *Ddit3* | NM_007837 | GCAGCGACAGAGCCAGAATA | CAAGGTGAAAGGCAGGGACT |  |
| *Hspa5* | NM_022310 | GTGTGTGAGACCAGAACCGT | GCAGTCAGGCAGGAGTCTTA |  |
| *Edem1* | NM_026041 | CAAGTGTGGGTACGCCACG | AAAGAAGCTCTCCATCCGGTC |  |
| *Blos1* | NM_013886 | CAAGGAGCTGCAGGAGAAGA | CCAGGAGGGTGAAGTAAGAGG |  |
| *Col61a* | NM_009933 | TGCTCAACATGAAGCAGACC | TTGAGGGAGAAAGCTCTGGA |  |
| *Atf4* | NM_009716 | CCTATAAAGGCTTGCGGCCA | GCTGGATTTCGTGAAGAGCG |  |
| *Ucp1* | NM_009463 | CACCTTCCCCCTGGACACT | CCCTAGGACACCTTTATACCT |  |
| *Prdm16* | NM_001277052 | AGGAGGAGGAGAGAGATTCCG | GTCCGGGTCAGGTTCATACAT |  |
| *Ppargc1a* | NM_008904 | CCCTGCCATTGTTAAGACC | TGCTGCTGTTCCTGTTTTC |  |
| *Cidea* | NM_009369 | ATCACAACTGGCCTGGTTACG | TACTACCCGGTGTCCATTTCT |  |
| *Cox5b* | NM_007752 | TGCTACCTCCAAAGGCAGCTTC | CATCGCTGACTCTCGCCTTTGT |  |
| *Cox7a1* | NM_022025 | CAGCGTCATGGTCAGTCTGT | AGAAAACCGTGTGGCAGAGA |  |
| *Cox8b* | NM_009944 | GAACCATGAAGCCAACGACT | GCGAAGTTCACAGTGGTTCC |  |
| *Ppara* | NM_011144 | TTCCCTGTGAACTGACGTTT | CCACCATGTTGGATGGATGTG |  |
| *Dio2* | NM_010050 | AGAGTGGAGGCGCATGCT | GGCATCTAGGAGGAAGCTGTT |  |
| *Adrb1* | NM_007419 | CCGAAAGCAGGTGAATGCAA | AGCCAGTAAGCCATACTAAGCCACA |  |
| Adrb2 | NM_009715 | CATTGATGTGTTGTGCGTCA | ACTCGGGCCTTATTCTTGGT |  |
| Adrb3 | NM_007420 | CCTTCCGTCGTCTTCTGTGT | AGCCATCAAACCTGTTGAGC |  |
| *Tnfa* | NM_013693 | CTGAACTTCGGGGTGATCGG | CTACGACGTGGGCTACAGG |  |
| *Il1b* | NM_008361 | TGCCACCTTTTGACAGTGATG | TGATGTGCTGCTGCGAGATT |  |
| *Il6* | NM_031168 | CACTTCACAAGTCGGAGGCT | CTGCAAGTGCATCATCGTTGT |  |
| *Ccl2* | NM_011333 | AGGTCCCTGTCATGCTTCTG | TCTCCAGCCTACTCATTGGGA |  |
| *Mgl1* | NM_021357 | CAGATCTGGGGCCGTCAAG | GGGAGGAAATGCATCTGGGT |  |
| *Il10* | NM_010548 | AGGCGCTGTCATCGATTTCT | ATGGCCTTGTAGACACCTTGG |  |
| *Adgre1* | NM_010130 | TCTGCTTCTGTACAGCCACG | CCTCAGAACCCACAGTGTCC |  |
| *Cd68* | NM_009853 | TGTTCAGCTCCAAGCCCAAA | GTACCGTCACAACCTCCCTG |  |
| *Abca1* | NM_013454 | TAGCAGCACCGTGTCTTGTC | GCGTGTCACTTTCATGGTCG |  |
| *Cd36* | NM_001159558 | TCCAGCCAATGCCTTTGC | TGGAGATTACTTTTCAGTGCAGAA |  |
| *Plin2* | NM_007948 | TCTGCGGCCATGACAAGTG | GCAGGCATAGGTATTGGCAAC |  |
| *Atp6v1b2* | NM_025989 | AGCCTCGTCTCACCTACAAGA | CTCAGCGTATCTGGGAAACTT |  |
| *Atp6v0d2* | NM_001289445 | AGCCAGCCTAACTCAGC | GCTTCTTCCTCATCTCCGTGTC |  |
| *Lamp2* | NM_010685 | ATGTGCCTCTCTCCGGTTAAA | GCAAGTACCCTTTGAATCTGTCA |  |
| *Lipa* | NM_008519 | GGAAACAGCAGAGGAAACACCT | CACGGGAGCCAAGACTAAAAC |  |
| *Fabp4* | NM_024406 | GGGGCCAGGCTTCTATTCC | GGAGCTGGGTTAGGTATGGG |  |
| *Fabp5* | NM_001272613 | ACGGCTTTGAGGAGTACATGA | CTCGGTTTTGACCGTGATG |  |
| *Ctsb* | NM_007798 | CAATGGCCGTTGAATGCACA | TGGTGTATGGTAAGCAGTG |  |
| *Trem2* | NM_031254 | AAAGCTAGCATGGAACCTCTC | TTGAATTCGAGCTCTCTAGACGT |  |
| *Cd9* | NM_007657 | TGGGGCTATACCCACAAGGA | GCTTTGAGTGTTTCCCGCTG |  |
| *Pepck* | NM_011044 | TGAAAGGCCGCACCATGTAT | GGGCGAGTCTGTCAGTTCAA |  |
| *G6p* | NM_008061 | ATCCGGGGCATCTACAATG | TGGCAAAGGGTGTAGTGTCA |  |
| *Acc* | NM_133360 | GCCTCTTCCTGACAAACGAG | TGACTGCCGAAACATCTCTG |  |
| *Scd1* | NM_009127 | GAGGCCTGTACGGGATCA | GCCCAGTCGTACACGTCA |  |
| *Fasn* | NM_007988 | GCTGGCATTCGTGATGGAGTCGT | AGGCCACCAGTGATGATGTAACTCT |  |
|  |  |  |  |  |
